# The time option of IVIG treatment is associated with therapeutic responsiveness and coronary artery abnormalities but not with clinical classification in the acute episode of Kawasaki disease

**DOI:** 10.1186/s12969-019-0352-3

**Published:** 2019-07-31

**Authors:** Sama Samadli, Fei Fei Liu, Goshgar Mammadov, Jing Jing Wang, Hui Hui Liu, Yang Fang Wu, Huang Huang Luo, Yue Wu, Wei Xia Chen, Dong Dong Zhang, Wei Wei, Peng Hu

**Affiliations:** Department of Pediatric, The First Affiliated Hospital of Anhui Medical University, No. 218 Ji-Xi Road, Hefei, 230022 People’s Republic of China

**Keywords:** American Heart Association, C-reactive protein, Coronary artery abnormalities, Intravenous immunoglobulin, Kawasaki disease

## Abstract

**Background:**

In the last decade, incomplete Kawasaki disease (KD), intravenous immunoglobulin (IVIG) non-response and coronary artery abnormalities (CAA) have experienced the increasing trends in China. In addition, the enhancement of pediatricians’ awareness may also raise the diagnostic rate of incomplete KD and stimulate more aggressive initial therapy in the acute episode of KD. Given this background, we hypothesize that the time option of IVIG treatment should be in parallel with peak time of systemic inflammation; either earlier or later IVIG treatment may affect the clinical classification, therapeutic responsiveness and CAA occurrence in KD patients. Therefore, the major objective of the present study is to identify whether the time option of IVIG treatment could be associated with the clinical classification, therapeutic responsiveness and CAA occurrence in the acute episode of KD.

**Materials and methods:**

A total of 153 children with KD were recruited between July 2015 and May 2018. All patients received the standard therapy of KD, including a single infusion of IVIG (2 g/kg) and aspirin (30–50 mg/kg/d). Blood samples were collected from all subjects within 24 h pre-IVIG treatment, respectively. Echocardiography was performed during the period from 2 days to 14 days after IVIG treatment.

**Results:**

(1) The clinical classification presented no significant heterogenicity among different treatment time (*x*^*2*^ = 1.59, *p* > 0.05) (2) Eleven KD patients resisted to IVIG treatment and 7 of them (63.60%) received the initial IVIG dose on day 5 and 6. (3) The distribution of CAA onset was subjected to a significant difference according to timing option of IVIG treatment (*x*^*2*^ = 11.94, *p* < 0.05).

**Conclusions:**

The time option of IVIG treatment is associated with therapeutic responsiveness and CAA but not with clinical classification in the acute episode of KD.

## Background

Kawasaki disease (KD) is an acute self-limited vasculitis of childhood that leads to coronary artery abnormalities (CAA) in approximate 5% of treated cases [1]. It is the leading cause of acquired heart disease in children younger than 5 years of age in Asian countries. According to a latest epidemiological study in Shanghai, China, the average annual incidence rate of KD was 50.5 per 100,000 children during the period of 2008–2012 [2]. In light of the 2017 American Heart Association (AHA) guidelines, the diagnosis criteria of KD include fever ≥5 days and four or more of the five major clinical features. Besides, incomplete KD should be considered in any case with persistent unexplained fever, fewer than 4 of the major clinical features, and compatible laboratory or echocardiographic findings [3]. Elevation of acute-phase reactants such as erythrocyte sedimentation rate (ESR) and C-reactive protein (CRP) is nearly universal and very helpful to reflect disease severity. Song et al. [4] evaluated the diagnostic efficiency of ESR and CRP in 67 children with persistent fever and found that the estimated sensitivity of ESR for predicting KD was 93.9%, and specificity was 83.3%; the estimated sensitivity of CRP for predicting KD was 69.0%, and specificity was 72.7%. Echocardiography is the primary imaging modality for cardiac assessment because it is noninvasive and has a very high sensitivity (100%) and specificity (95–100%) for the detection of abnormalities of the proximal coronary artery segments [5].

Intravenous immunoglobulin (IVIG) combined with high-dose aspirin is the first choice for suppressing systemic inflammation and preventing CAA. In our latest study, white blood cells counts (WBC), absolute neutrophil counts (ANC), CRP and procalcitonin (PCT) levels markedly increased in the acute episode of KD, whereas declined to 30%~ 90% after IVIG treatment [1]. On the other hand, a multi-center, randomized trial from USA documented that timely initiation of IVIG treatment could reduce the incidence of CAA from 20 to 6.8% at the two-week visit [6]. Nevertheless, patients with a delayed diagnosis may still be candidates for IVIG treatment. Qiu et al. [7] analyzed inflammatory mediators and risk factors for CAA in 59 KD children who received IVIG treatment > 10 days and found that a delayed IVIG treatment may contribute to the higher levels of CRP and ESR, and serve as an independent risk factor for the development of CAA (adjusted OR = 2.90, 95%CI = 1.42, 5.91). Despite IVIG plus high-dose aspirin is considered as the first-line treatment, some KD patients develop recrudescent or persistent fever more than 36 h after the end of IVIG infusion and are termed IVIG non-response [3]. In this condition, another 2 g/kg dose of IVIG plus a corticosteroid is usually advocated [8].

Several previous epidemiologic surveys have suggested the onset of incomplete KD experiences an increasing trend in China. A long-term retrospective study from Inner Mongolia revealed that the incidence of incomplete KD increased from 10.38% in 2003 to 40.12% in 2012 [9]. In the last decade, the incidence of IVIG non-response ranged from 4.9 to 17.8% [2, 10, 11]. Based on several epidemiological surveys from mainland China, the overall trend in CAA occurrence also appeared to be on the rise from 15.9 to 63.3%, far higher than the other Asian countries [2, 12, 13]. Several published scoring systems have revealed that age in months, CRP and ESR were associated with incomplete KD; day of illness at initial treatment, age in months, percentage of neutrophils, platelet count (PLT), serum aspartate aminotransferase, sodium and CRP served as independent predictors of IVIG non-response; male, fever duration, albumin, percentage of eosionphils and monocytes predicted CAA risks [11, 14–16]. In addition, the enhancement of pediatricians’ awareness may raise the diagnostic rate of incomplete KD and stimulate more aggressive initial therapy in the acute episode of KD. Given this background, we hypothesize that the time option of IVIG treatment should be in parallel with peak time of systemic inflammation; either earlier or later IVIG treatment may affect the clinical classification, therapeutic responsiveness and CAA occurrence in KD patients. Therefore, the major objective of the present study is to identify whether the time option of IVIG treatment could be associated with the clinical classification, therapeutic responsiveness and CAA occurrence in the acute episode of KD.

## Materials and methods

### Patient selection

The observational period of our study covered the time from July 2015 to May 2018. Therefore, the sample size was completely dependent on the total number of KD patients admitted to our center in this period. After retrospectively reviewing all the medical records, 153 KD patients were involved eventually. Approval for this study was acquired from the Medical Ethic Committee of Anhui Medical University and informed consent was obtained from all parents before study entry. According to AHA guidelines [17], the diagnosis of complete KD is based on the presence of ≥5 days of fever and ≥ 4 of the following five signs: (1) bilateral conjunctival injection without exudates; (2) changes in the oral mucosa, such as erythema and cracking lips, erythema of the pharynx, strawberry tongue; (3) changes in extremities, such as redness and swelling in the acute phase, periungual desquamation in the subacute phase; (4) polymorphous exanthema; (5) cervical lymphadenopathy, (≥1.5 cm in diameter), usually unilateral. Patients with fever for ≥5 days and at least 2 of the principal features were diagnosed as incomplete KD, if no other disease processes could explain the illness. All patients received the standard therapy of KD, including a single infusion of IVIG (2 g/kg) and aspirin (30–50 mg/kg/d). IVIG-nonresponsive KD was defined as persistent or recrudescent fever ≥36 h after completion of the initial IVIG infusion [17].

### Laboratory analysis

Blood samples were collected from all subjects within 24 h pre-IVIG treatment, respectively. Venous blood (2 ml) was collected in a gel coagulation-promoting vacuum tube and centrifuged immediately at 2800 g for 15 min at room temperature, and plasma samples were stored at − 80 °C. WBC, ANC and PLT were performed using a flow cytometer (Sysmex XE-2100). ESR and CRP were determined by the Westergren method and immunoturbidimetry respectively.

### Echocardiography

Echocardiography was performed during the period from 2 days to 14 days after IVIG treatment. Children unable to cooperate were sedated according to local practice. CAA is defined as a coronary artery having an internal diameter of at least 3 mm in Children < 5 years or at least 4 mm in children ≥5 years; or a segment with an internal diameter at least 1.5 times larger than that of an adjacent segment by echocardiogram [17]. Echocardiographic results were interpreted independently by 2 echocardiographers who were blinded to treatment assignment. A third reading was performed if necessary to resolve discordant interpretations.

### Statistical analysis

The collected data were stratified with respect to the time of IVIG treatment. Normally distributed continuous data were expressed as mean ± standard deviation. Comparisons of the frequencies between groups were analyzed using Pearsons’ chi-square tests. Comparison of mean values between groups was carried out using the independent sample t-test. Comparison of mean values among groups was carried out using one-way ANOVA and post hoc analysis was calculated using the Student-Newman-Keuls test. Statistical significance was identified with a *p* value < 0.05. Statistical analysis was performed using the statistical package for social studies SPSS version 16.0.

## Results

### Demographic features

In the present study, 85 males and 68 females were diagnosed as having KD, with a mean age of 35.22 ± 30.22 months and a range from 3 months to 13 years

#### Clinical classification

All 5 classic diagnostic criteria for KD were met in 48 cases (31.37%), 4 criteria in 60 (39.22%), 3 criteria in 25 (16.34%), and 2 criteria in 20 (13.07%). Therefore, 108 children (70.59%) had complete KD, including 64 males and 44 females with the mean age of 36.43 ± 30.52 months; 45 children (29.41%) had incomplete KD, including 21 males and 24 females with the mean age of 32.33 ± 29.64 months. The mean age and male/female ratio were almost identical between different clinical phenotypes (*p* > 0.05).

#### IVIG treatment

Among 153 KD patients, 11 of them (7.19%) were identified as IVIG-nonresponsive KD and had persistent fever about 71.00 ± 12.27 h after IVIG treatment, including 4 males and 7 females with the mean age of 33.73 ± 27.46 months. In contrast, 142 patients were diagnosed with IVIG-responsive KD and exhibited a dramatic decrease in fever duration after IVIG treatment (8.13 ± 7.04 h, *t* = 8.470, *p* < 0.05), including 81 males and 61 females with the mean age of 35.34 ± 30.51 months.

#### Coronary artery involvement

According to the enlarged internal diameter of coronary artery, 10 KD patients (6.54%) were defined as having coronary arteritis during the whole observational period (left coronary artery: 3.34 ± 0.42 mm; right coronary artery: 2.93 ± 0.97 mm), including 7 males and 3 females with the mean age of 28.50 ± 23.96 months. In contrast, 143 patients (93.46%) had normal coronary artery after IVIG treatment (left coronary artery: 2.09 ± 0.37 mm, right coronary artery: 2.01 ± 0.33 mm), including 78 males and 65 females with the mean age of 35.69 ± 30.02 months.

### Inflammatory mediators

Inflammatory mediators in patients with different types of KD are presented in Table 1. Plasma WBC was significantly increased in patients with IVIG-nonresponsive KD when compared with their IVIG-responsive counterparts (*t* = 2.000, *p* < 0.05); however, no significant differences in ANC, PLT, CRP and ESR were observed between the 2 groups (*p* > 0.05). Regardless incomplete KD and coronary arteritis existed or not, no significant differences were observed in WBC, ANC, PLT, CRP and ESR (*p* > 0.05).

**Table 1:** Inflammatory mediators in patients with different types of KD

KD patients (*n* = 153)	WBC (× 10^9^/l)	ANC (× 10^9^/l)	PLT (× 10^9^/l)	CRP (mg/l)	ESR (mm/h)
Clinical classification					
Complete (*n* = 108)	12.96 ± 4.77	10.84 ± 11.59	355.71 ± 137.71	56.64 ± 46.57	60.39 ± 25.02
Incomplete (*n* = 45)	14.58 ± 6.17	12.11 ± 14.52	349.93 ± 96.51	65.75 ± 44.67	64.65 ± 22.51
IVIG treatment					
Response (*n* = 142)	13.22 ± 4.98	11.11 ± 12.81	351.62 ± 124.09	59.24 ± 47.26	60.79 ± 24.28
Nonresponse (*n* = 11)	16.24 ± 7.72*	12.47 ± 7.37	384.91 ± 160.52	60.31 ± 27.53	72.64 ± 22.95
CA involvement					
Normal CA (*n* = 143)	13.49 ± 5.29	11.45 ± 12.85	352.01 ± 123.34	60.37 ± 46.81	62.05 ± 23.91
Coronary arteritis (n = 10)	12.68 ± 4.95	7.77 ± 3.94	382.70 ± 173.54	44.28 ± 31.49	55.80 ± 30.41

* *p* < 0.05

**Fig. 1:**
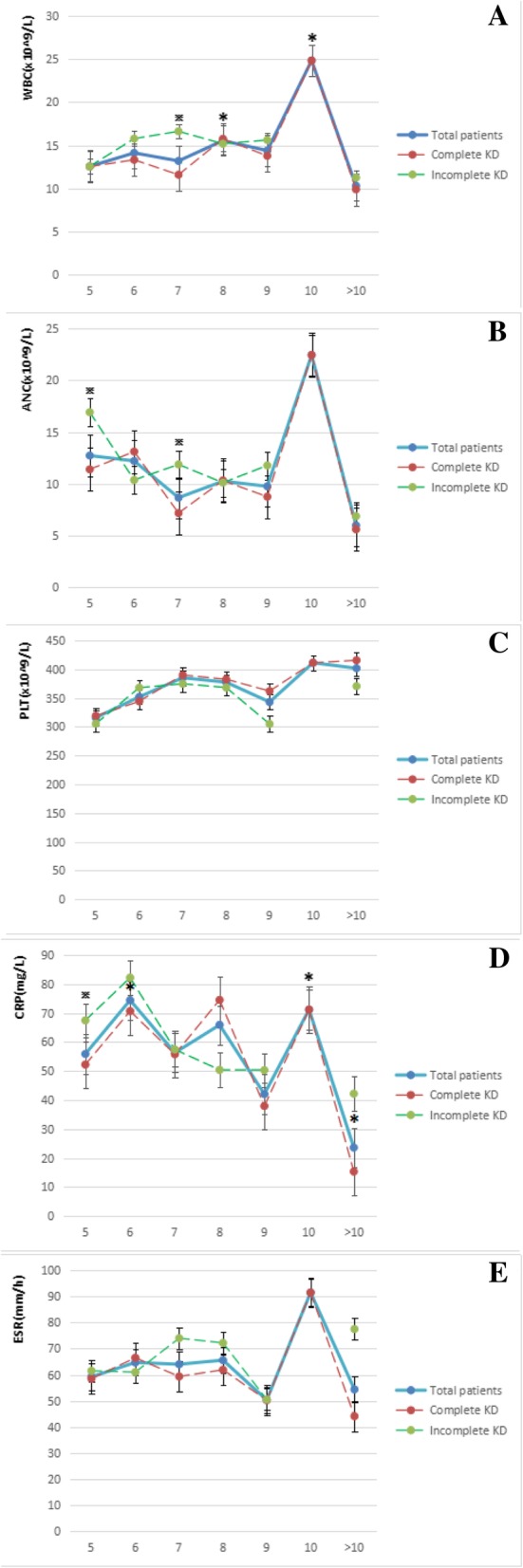
The distribution of inflammatory mediators among different treatment time. *p < 0.05, inflammatory mediators increased time-dependently and reached statistical significance. ^※^*p* < 0.05, inflammatory mediators significantly increased in the incomplete group. **a** WBC increased time-dependently and reached statistical significance on day 8 and day 10 when comparing with day 5 (*p < 0.05). WBC significantly increased in the incomplete group on day 7 (^※^*p* < 0.05). **b** ANC significantly increased in the incomplete group on day 5 and day 7 (^※^*p* < 0.05). **d** CRP increased time-dependently and reached statistical significance on day 6, day 10 and after 10 days when comparing with day 5 (*p < 0.05). CRP significantly increased in the incomplete group on day 5 (^※^*p* < 0.05). There were no significant differences were found in PLT (**c**) and ESR (**e**) among different treatment time

Inflammatory mediators among different treatment time are illustrated in Fig. 1. As disease progression, the plasma levels of WBC, ANC, PLT, CRP and ESR markedly increased in the acute phase of KD. In the total patients, WBC, ANC, CRP and ESR reached the largest values on day 10, and significant differences were observed in WBC and CRP among different treatment time (*F* = 3.418, *p* < 0.05; *F* = 2.545, *p* < 0.05); more specifically, WBC increased time-dependently and reached statistical significance on day 8 (*p* < 0.05) and day 10 (*p* < 0.05) when comparing with day 5, and CRP also increased and reached statistical difference on day 6 (*p* < 0.05) and day 10 (*p* < 0.05) when comparing with day 5. To probe the influences of clinical classifications to inflammatory mediators, the total patients were divided into the complete KD group and the incomplete KD group at each treatment time. Compared with the complete KD group, WBC significantly increased in the incomplete group on day 7 (*p* < 0.05), ANC significantly increased in the incomplete group on day 5 and day 7 (*p* < 0.05), and CRP significantly increased in the incomplete group on day 5 (*p* < 0.05).

### Timing option of IVIG treatment & clinical classification

Pearsons’ chi-square test was conducted to observe the association of IVIG treatment with clinical classification Table 2. According to the timing option of IVIG treatment, 140 KD patients (91.50%) received the initial IVIG treatment within 10 days of illness, including 50 cases (32.68%) on day five, 46 cases (30.06%) on day six, 22 cases (14.38%) on day seven, 14 cases (9.15%) on day eight, 6 cases (3.92%) on day nine, and 2 cases (1.31%) on day ten; in addition, there were still 13 cases (8.50%) receiving the initial IVIG treatment after 10 days of illness. However, the clinical classification presented no significant heterogenicity among different treatment time (*x*^*2*^ = 1.59, *p* > 0.05), thus the earlier IVIG treatment appeared not to relate to the onset of incomplete KD.

**Table 2:** The association of treatment time with clinical classification

Time option of IVIG	Complete KD (*n* = 108)	Incomplete KD (*n* = 45)	*x* ^*2*^ *p*
5th day	38	12	1.59 0.92
6th day	31	15	
7th day	15	7	
8th day	9	5	
9th day	4	2	
10th day	2	0	
>10th day	9	4	

### Timing option of IVIG treatment & CAA

In the present study, 10 KD patients were defined as having CAA in the acute stage. According to the timing option of IVIG treatment, 7 of them received the initial IVIG treatment within 10 days of illness, including 2 cases on day five, 2 cases on day six, 1 case on day eight and 2 cases on day nine; in addition, there were still 3 cases receiving the initial IVIG treatment after 10 days of illness. The distribution of CAA onset was subjected to a significant difference according to timing option of IVIG treatment (*x*^*2*^ = 11.94, *p* < 0.05).

## Discussion

IVIG is a well-established standard therapy for KD and effectively reduces systemic inflammation [3]. Furthermore, IVIG treatment is effective in shortening the length of hospital stay and preventing undesirable cardiac events in KD patients. Klassen et al. [18] compared the length of hospital stay and cost effectiveness in 100 KD patients with different treatment strategies, and discovered that the hospitalization time (3 days) was significantly shorter and the total medical expense ($118,200) was significantly lower in the IVIG high-dose group than those in the aspirin alone group (10 days and $323,400). In another retrospective study, Bal et al. [19] identified the risks for development and delay in resolution of CAA in association with IVIG administration within or after 10 days of KD onset. The risk for CAA was significantly lower among these patients admitted within 10 days (OR = 3.1) in comparison with their counterparts received IVIG after 10 days (OR = 5.3); and the resolution time of CAA was significantly shorter among these patients admitted within 10 days than their counterparts (6 months vs.12 months). In the present study, we observed that the duration of fever decreased dramatically after the initial IVIG treatment in 142 KD patients, which was mainly attributed to the depressed systemic inflammation. Besides, the potential mechanisms of IVIG treatment may include several immunoregulative processes. Lau et al. [20] established a murine model of KD to examine the effect of IVIG, and showed that IVIG inhibited T cell proliferation, tumor necrosis factor-α production and nuclear factor (NF) -κB activation in a dose-dependent manner, all of which are critical steps preventing coronary artery damage.

Although IVIG is highly effective in KD, approximately 10 to 20% of KD patients develop recrudescent or persistent fever at least 36 h after the end of their IVIG infusion [21]. To date, the immunologic basis of IVIG non-response remains unknown. Several studies have documented that the single-nucleotide polymorphisms in STX1B and carcinoembryonic antigen-related cell adhesion molecule 1 play a vital role in IVIG non-response [22, 23]. In the present study, 11 KD patients resisted to IVIG treatment and 7 of them (63.60%) received the initial IVIG dose on day 5 and 6. Similarly, a multi-institutional, retrospective cohort study from Japan indicated that the early treatment group (on day 4) had a significantly higher rate of IVIG non-response than the conventional treatment group [24]. Therefore, earlier intervention before peak time of systemic inflammation may contribute to IVIG non-reponse.

As outlined in the 2017 AHA guidelines [3], KD is accompanied by the gradual elevations of WBC, ANC, CRP and ESR time-dependently in the acute stage. Consistently, the present study also showed that WBC, ANC, CRP and ESR reached the largest values on day 10. In California, Tremoulet et al. [25] performed a retrospective chart review of 380 KD patients and found that ANC and CRP peaked within the first 10 days of illness, whereas PLT peaked between day 11 and day 20. Lee et al. [26] evaluated the inflammatory mediators according to the fever duration in 152 Korean children with KD and discovered that WBC, ANC and CRP reached their summits on day 6, earlier than our findings. Therefore, understanding the dynamic changes in laboratory parameters may assist pediatricians in evaluating the inflammatory status of KD patients.

The presence of fever for ≥5 days with 4 of the 5 other principal features fulfills the diagnosis of complete KD, whereas the above criteria unfortunately do not identify all children with the illness. According to the current available data, approximately 20 to 40% of patients are diagnosed with incomplete KD [27, 28]. In this study, 45 patients developed incomplete KD (29.40%) and 27 of them (60.00%) received the initial IVIG dose on day 5 and 6. However, the clinical classification presented no significant heterogenicity among different treatment time, thus early recognition to incomplete KD seemed to be relatively challenging. Identically, Sittiwangkul et al. [29] analyzed the medical records of 170 KD patients in Thailand from 2000 to 2008 and found that timing option of IVIG treatment was not associated with the onset of incomplete KD. In contrast, another clinical survey from Wenzhou [7], China revealed that the proportion of incomplete KD in the delayed therapy group (received IVIG treatment > 10 days) was significantly higher than in the conventional therapy group (received IVIG treatment ≤10 days).

CAA serves as a predictor to the long-term prognosis of KD. Currently, several inflammatory mediators, such as NF-κB, interleukin (IL)-1β, IL-6, fibroblast growth factor-23 and transforming growth factor-β, have been reported to participate in CAA onset [30–32]. Besides, a growing body of evidence have shown that SLC8A1, male, infants < 6 months old, low serum albumin, high ESR, CRP, mycoplasma infection, IVIG started after the 10th day of illness and IVIG non-responders may increase the risk of CAA [33–35]. In the present study, the distribution of CAA onset was subjected to a significant difference according to timing option of IVIG treatment; a subsequent usage of IVIG may result in a higher occurrence of CAA and a more severe vasculitis requires more aggressive therapy. More persuasively, according to the 20th nationwide survey of KD in Japan [36], CAA incidence during the convalescent phase was significantly higher in the late IVIG treatment group (≥10 days) than those who received IVIG treatment within day 10. Therefore, the early treatment of IVIG is considered to be effective for suppressing systemic inflammation and preventing CAA.

## Conclusions

The time option of IVIG treatment is associated with therapeutic responsiveness and CAA but not with clinical classification in the acute episode of KD.

## Data Availability

The datasets generated and/or analysed during current study are available from the corresponding author on reasonable request.

## Abbreviations

AHA: American Heart Association
ANC: absolute neutrophil counts
CAA: coronary artery abnormalities
CRP: C-reactive protein
ESR: Erythrocyte sedimentation rate
IVIG: Intravenous immunoglobulin
KD: Kawasaki disease
PCT: Procalcitonin
WBC: White blood cells counts
